# Evaluation of increasing levels of mycotoxin-containing corn fines and mitigants on nursery pig growth performance

**DOI:** 10.1093/tas/txaf025

**Published:** 2025-02-18

**Authors:** Duncan B Paczosa, Tyler B Chevalier, Sunday A Adedokun, Lan Zheng, Merlin D Lindemann

**Affiliations:** Department of Animal and Food Sciences, University of Kentucky, Lexington, KY 40546, USA; Department of Animal and Food Sciences, University of Kentucky, Lexington, KY 40546, USA; Department of Animal and Food Sciences, University of Kentucky, Lexington, KY 40546, USA; DSM-Firmenich, Plainsboro, NJ 08536, USA; Department of Animal and Food Sciences, University of Kentucky, Lexington, KY 40546, USA

**Keywords:** clinical chemistry, fumonisin, mycotoxins, pigs

## Abstract

The effects of feeding corn fines (screenings) containing mycotoxin levels greater than the FDA guidance (fumonisins) and advisory (deoxynivalenol) levels were evaluated using 150 crossbred pigs (initial BW: 6.42 ± 0.06 kg; 90 barrows and 60 gilts) in an 8-wk study by adding contaminated corn fines to create six diets. The corn fines used contained prestudy analyzed mycotoxin levels of 20,334 ppb total fumonisin, 1,499 ppb zearalenone, and 5,075 ppb total deoxynivalenol. The corn fines were added into a corn-soybean meal basal diet at 0%, 20%, 40%, and 60% corn fines (Diets 1 to 4, respectively). Diet 5 was created by adding 40 ppm of boron (as sodium tetraborate decahydrate, 11.34% B) to Diet 4. Diet 6 was created by adding 0.25% Biofix Plus with FUMzyme (BPF; dsm-firmenich, Plainsboro, NJ) to Diet 4. Dietary treatments were fed for 6 wk; after that, all pigs received a common corn-soybean meal basal diet without fines for about 2 wk. The lightest, median, and heaviest pigs in a pen were selected at week 3, and serum was collected from those pigs on weeks 3, 6, and 8. Serum clinical chemistry and sphinganine:sphingosine ratio (SA:SO) were determined at week 6. Increasing fines linearly decreased ADG during weeks 1–6 (*P* = 0.03). Comparing Diets 4 and 5 to Diet 1 during weeks 1–6, there was a decrease in ADG (*P* < 0.05); subsequently, the difference in Diets 4 and 5 compared to Diet 1 was no longer significant for weeks 1–8. Comparing Diet 6 to Diets 1 and 4 during weeks 1–6, pigs fed Diet 6 were able to recover 57% of the lost ADG that occurred when corn fines were increased from 0% to 60%. During week 7–8 (all pigs on a common diet), pigs on Diets 5 and 6 had an increase in ADG compared to Diet 1. SA:SO linearly increased as fines in the diet increased (Diets 1 to 4; *P* = 0.001), but the addition of BPF ameliorated 95% of this increase. In conclusion, as mycotoxins increased, pigs exhibited negative effects in ADG, but the additive Biofix Plus with FUMzyme ameliorated a portion of these effects. Further, the recovery from week 7–8 from all pigs does show the importance of feeding clean corn to optimize ADG, ADFI, and gain/feed ratio.

## Teaser

The addition of mycotoxin fines up to 60% of the diet resulted in a detriment to pig performance. The feed additive Biofix Plus with FUMzyme ameliorated a portion of the lost pig performance that had resulted from the addition of mycotoxins in the diet.

## INTRODUCTION

Regulatory limits or maximum levels have been established for a few mycotoxins separately by the FDA and the EU in feeds and foods. A US corn mycotoxin report (dsm, 2024) analyzed over 250 corn samples from the 2023 harvested crop from 20 states for 6 major mycotoxin groups and reported that 94% of the samples were above the detection limit for at least one toxin and that 78% contained more than 1 detectable mycotoxin. Weaver et al. (2021) also reported that out of 711 corn grain samples tested, 90.2% tested positive for more than one mycotoxin. Cargill (2022) similarly report that two or more mycotoxins tested positive in 95% of 311,009 samples of corn, cereals, oilseeds, and other feedstuffs (silage, oils, beans, beets, potatoes). Thus, mycotoxins are clearly common in corn and, therefore, would be expected in pig feed at different concentrations and occurrences. Additionally, simultaneous exposure to mycotoxins can result in additive, synergistic, or antagonistic effects (Alassane-Kpembi et al., 2017). This may cause mycotoxicosis symptoms to increase drastically (synergistic), remain as expected (additive), or be suppressed (below expected). Increased awareness of the economic loss from mycotoxins has resulted in an increase in research for possible mycotoxin mitigants. Previous research evaluating potential mitigants in diets contaminated with aflatoxin (Lindemann et al., 1993; Schell et al., 1993a, 1993b) or with deoxynivalenol (Frobose et al., 2015) has demonstrated improvement in performance with some dietary additives.

Paczosa et al. (2025) demonstrated that pigs can detect differing concentrations of mycotoxins in diets when given a choice of diets and that they will choose the diet with lower levels of mycotoxins; however, when not given a choice of those same diets, there is not necessarily a difference in growth performance. This research was conducted as a follow-up to Paczosa et al. (2025). The levels of corn fines used to supply the mycotoxins in Paczosa et al. (2025) were supplied at 20% of the diet and were not high enough to cause a detriment in the performance response measures within the 3 wk time frame of the study. The same corn fines (contaminated with fumonisins [FUM], deoxynivalenol [DON], and zearalenone [ZEA] with FUM being the principal contaminant) and performance response measures were investigated herein, but the amount of those fines was increased from 20% up to 60%, and the duration of the study was increased from 3 wk to 6 wk. Additionally, two possible mitigants were used in the present study. Because adverse effects of mycotoxins would follow a dose/time relationship, it was hypothesized that by increasing the dosage of the fines/mycotoxins and increasing the amount of time that the pigs were fed the diets, that negative effects in growth performance would be observed. The objective of this study was to increase the time-dose aspects of the study in an attempt to create a clear growth detriment caused by these mycotoxins and then to determine if these potential mitigants might ameliorate any detriments. Additionally, assuming a detriment would be observed, blood samples were taken from multiple pigs within each pen to assess normal clinical chemistry analytes as potential biomarkers of mycotoxin-related performance detriments.

## MATERIALS AND METHODS

This experiment was conducted in an environmentally controlled room at the University of Kentucky Swine Research Center. The experiment was conducted under protocols approved by the Institutional Animal Care and Use Committee of the University of Kentucky and within the husbandry guidelines for the care and use of agricultural animals in research and teaching (Ag Guide, 2020).

### Diets

Corn harvested at the University of Kentucky Research Farm from the 2020 crop year was screened by a grain cleaner to remove corn fines that would contain the highest concentrations of mycotoxins. The fines used to mix the 6 diets in this study were the same batch of fines that were used in Paczosa et al. (2025). Experimental diets were formulated to meet or exceed the NRC (2012) daily requirement estimates based on pig BW (Table 1). The corn fines used contained mycotoxin levels that were analyzed prestudy by North Dakota State University Veterinary Diagnostic Laboratory (Fargo, ND) to be 20,334 ppb total FUM, 1,499 ppb ZEA, and 5,075 ppb total deoxynivalenol. To evaluate the effects of feeding these corn fines (screenings) containing mycotoxin levels greater than the FDA cautionary levels, dietary corn fines were added to a corn-soybean meal-based basal diet at concentrations of 0%, 20%, 40%, and 60% of the diet by simply replacing the regular ground corn with the corn fines (Diets 1 to 4, respectively). To minimize differences in non-treatment components of the diet, Diets 1 and 4 were mixed according to Table 1, with the first diet serving as the control diet (0% fines) and the second diet serving as Diet 4 (60% fines). Diet 4 was derived simply by replacing an equal amount of clean corn with corn fines. By blending appropriate amounts of Diets 1 and 4, intermediate diets (20% and 40% fines) were created.

**Table 1.** Composition of the basal diet (%, as-fed basis)

	Weeks 1–6	Weeks 1–6	Weeks 7–8
Ingredient, %	Diet 1	Diet 4	Common Diet
Corn	60.00	0.00	69.80
Corn fines	0.00	60.00	0.00
Soybean meal	34.55	34.55	25.00
Grease	2.00	2.00	2.00
L-Lysine	0.28	0.28	0.25
DL-Methionine	0.19	0.19	0.10
L-Threonine	0.11	0.11	0.08
Dicalcium phosphate	1.20	1.20	1.10
Limestone	0.90	0.90	0.90
Salt	0.50	0.50	0.50
Copper sulfate	0.08	0.08	0.08
Trace-mineral premix^1^	0.10	0.10	0.10
Vitamin premix^2^	0.08	0.08	0.08
Santoquin^3^	0.02	0.02	0.02
Total	100.00	100.00	100.00
Calculated nutrient composition^4^			
Metabolizable energy, kcal/kg	3361	3361	3371
Crude protein, %	21.89	21.89	18.04
SID Lysine, %^5^	1.24	1.24	0.98
Calcium, %	0.73	0.73	0.68
Total Phosphorus, %	0.62	0.62	0.56
STTD Phosphorus, %^6^	0.39	0.39	0.35
Analyzed nutrient composition^7^		-	
Gross energy, kcal/kg	3963	3988	
Crude protein, %	19.94	21.09	-
Calcium, %	0.66	0.76	-
Total phosphorus, %	0.56	0.63	-

^1^Provided the following per kg of diet: Zn, 131 mg as ZnO; Fe, 131 mg as FeSO_4_·H_2_O; Mn, 45 mg as MnO; Cu, 13 mg as CuSO_4_·5H2O; I, 1.5 mg as CaI_2_O_6_; Co, 0.23 mg as CoCO_3_; Se, 0.28 mg as NaSeO_3_.

^2^Provided the following per kg of diet: vitamin A, 6,600 IU; vitamin D_3_, 880 IU; vitamin E, 44 IU; vitamin K (as menadione sodium bisulfite complex), 6.4 mg; thiamin, 4.0 mg; riboflavin, 8.8 mg; pyridoxine, 4.4 mg; vitamin B_12_, 33 μg; folic acid, 1.3 mg; niacin, 44 mg; pantothenic acid, 22 mg; D–biotin, 0.22 mg.

^3^Provided 130 mg ethoxyquin per kg of diet (Novus International, St. Louis, MO).

^4^Values for metabolizable energy, crude protein, SID lysine, calcium, total phosphorus, and STTD phosphorus were obtained from the feedstuff values listed in the NRC (2012).

^5^SID = standardized ileal digestible basis.

^6^STTD = standardized total tract digestible basis.

^7^All nutrients analyzed in duplicates.

Diets 5 and 6 were used to evaluate two potential dietary mycotoxin mitigants. Diets 5 and 6 were created by taking a portion of Diet 4 and adding the respective possible mitigant. Diets 5 and 6 consisted, thus, of 60% corn fines plus 40 mg B/kg diet (as sodium tetraborate decahydrate, 11.34% B) and 0.25% Biofix Plus with FUMzyme (BPF; dsm-firmenich, Plainsboro, NJ), respectively. Biofix Plus with FUMzyme is composed of bentonite, diatomaceous earth, yeast (saccharomyces cerevisiae) culture, kelp meal, natural flavoring compounds, and fumonisin esterase (FUMzyme^®^).

At the end of Week 6, all feeders were cleaned out, and all pigs were fed a common grower diet that contained no corn fines (Table 1).

### Animals, Experimental Design, and Housing

A total of 150 crossbred pigs (Yorkshire × Landrace × Large White; 90 barrows and 60 gilts; initial BW: 6.42 ± 0.06 kg) were blocked by sex and body weight (BW) and then randomly allotted within block to one of six dietary treatments using the Experimental Animal Allotment Program (Kim and Lindemann, 2007). Pigs were housed in totally-slatted nursery pens (1.22 × 2.44 m^2^) containing 5 pigs/pen with a total of 5 replicate pens. In total, the experiment lasted for 8 wk; during weeks 1–6 pigs were offered their respective experimental diet whereas during weeks 7 and 8 all pigs were offered a common grower diet to assess potential compensatory growth effects.

### Data Collection and Laboratory Analysis

Pig BW and feed disappearance were recorded every week to calculate average daily gain (ADG), average daily feed intake (ADFI), and gain to feed ratio (G:F). A total of 3 pigs per pen were selected at the end of week 3 by determining the smallest, heaviest, and median weight pigs within a pen for blood collection. Blood was collected from these same pigs at the end of weeks 6 and 8. Blood samples were collected via vena cava puncture using a needle holder and vacutainer tube with polymer gel (Becton, Dickinson, and Company, Franklin Lakes, NJ) for serum separation. Following blood collection, samples were allowed to clot at room temperature for 30 to 45 min. Serum was obtained by centrifugation of the blood samples at 2500 × g and 4 °C for 20 min. Serum samples were stored at −80 °C until the chemical assays were performed.

To determine serum clinical chemistry profiles, the serum from the 3 pigs per pen at the end of week 6 (n = 90) was selected for analysis. Aliquots of serum were sent to the University of Kentucky Veterinary Diagnostic Laboratory (Lexington, KY), where a porcine serum chemistry panel was performed using an Alfa Wassermann Vet Axel chemistry analyzer (Alfa Wassermann Diagnostic Technologies LLC., West Caldwell, NJ). The intent when taking samples at three time points was to analyze the samples taken at 6 wk (the longest exposure time), and if a treatment effect was observed, the 3-wk samples could be analyzed to see if the effect was starting to develop at that time point, and/or the 8 wk-samples could be analyzed to look at potential recovery.

Diet samples were analyzed for nitrogen (method 990.03; AOAC Int., 2006), calcium and phosphorus (method 993.14; AOAC Int., 2006) at the Experiment Station Chemical Laboratories at the University of Missouri (Columbia, MO). Gross Energy was analyzed using a bomb calorimeter (Parr adiabatic bomb calorimeter, model 6,200, Parr Instruments, Moline, IL) according to the manufacturer’s instructions, with the calibration standard being benzoic acid.

Samples of corn fines initially obtained at the feed mill following diet mixing were sent to the North Dakota State University Veterinary Diagnostic Laboratory (Fargo, ND) for mycotoxin analysis. The mycotoxin panel and the detection levels (DL) were: aflatoxin B_1_ (DL-20 ppb), aflatoxin B_2_ (DL-20 ppb), aflatoxin G1 (DL-20 ppb), aflatoxin G2 (DL-20 ppb), fumonisin B_1_ (DL-200 ppb), fumonisin B_2_ (DL-200 ppb), fumonisin B_3_ (DL-200 ppb), HT-2 toxin (DL-200 ppb), T2 toxin (DL-20 ppb), DAS (DL-200 ppb), ochratoxin A (DL-20 ppb), sterigmatocystin (DL-20 ppb), zearalenone (DL-100 ppb), deoxynivalenol (DL-200 ppb), 15-ADON (DL-200 ppb) and 3-ADON (DL-200 ppb).

Samples of corn, corn fines, and individual diets, were obtained at the feed mill following diet mixing. Individual diets, fines, and corn samples were sent to Romer Labs (Union, MO) for mycotoxin analysis. The mycotoxin panel and detection levels (DL) were aflatoxin B1 (DL-1.3 ppb), aflatoxin B2 (DL-1.2 ppb), aflatoxin G1 (DL-1.1 ppb), aflatoxin G2 (DL-1.6 ppb), fumonisin B_1_ (DL-100 ppb), fumonisin B_2_ (DL-100 ppb), fumonisin B_3_ (DL-100 ppb), HT-2 toxin (DL-100 ppb), T2 toxin (DL-100 ppb), diacetoxyscirpenol (DL-100 ppb), ochratoxin A (DL-1.1 ppb), deoxynivalenol (DL-100 ppb), acetyldeoxynivalenol (DL-100 ppb), nivalenol (DL-100 ppb), fusarenon X (DL-100 ppb), neosolaniol (DL-100 ppb), and zearalenone (DL-51.7 ppb).

Serum aliquots from two barrows and two gilts from Diets 1 to 4 and 6 were sent to the University of Missouri Veterinary Medical Diagnostic Laboratory (Columbia, MO), and analyzed for sphinganine/sphingosine by high performance liquid chromatography (HPLC) with fluorescence detection utilizing a modified method of Hsiao et al. (2007).

### Statistics

Data were analyzed as a randomized complete block design. The analysis of variance was conducted by using the General Linear Model (Proc GLM) procedure of the Statistical Analysis System (SAS, version 9.4, Cary, NC). Orthogonal polynomial contrasts were used to determine linear and quadratic effects with increasing dietary levels of corn fines in Diets 1 to 4 as well as preplanned single degree of freedom comparisons for Diets 4 vs. 5 and 5 vs. 6. Performance results utilized the pen as the experimental unit and clinical chemistry utilized the individual pig as the experimental unit with a level of significance set at *P* < 0.05.

## RESULTS AND DISCUSSION

### Diet Analysis

The corn fines used in this experiment were the same fines as used in Paczosa et al. (2025) but this current experiment occurred roughly 1 yr later. Paczosa et al. (2025) also outlines the process used to remove fines (highly concentrated in mycotoxins) from the corn grain. In Paczosa et al. (2025), the fines were analyzed at North Dakota State University Veterinary Diagnostic Laboratory (Fargo, ND) for mycotoxin concentrations. For this current study two mycotoxin testing labs were utilized, corn fines only were analyzed at North Dakota State University Veterinary Diagnostic Laboratory (Fargo, ND), and mycotoxin analysis at Romer Labs (Union, MO) was utilized for both corn fines and diets. Mycotoxin tests from NDSU reported concentrations of FUM, DON, and ZEA in corn fines for Paczosa et al. (2025) were 23,038, 5,032, and 1,446 ppb, respectively, compared to assay values for the same batch of fines for this experiment of 20,334, 5,075, and 1,499 ppb, respectively. Analyzed concentrations from Romer labs (Table 2) for the same fines were 16,100, 2,997, and 1,626 ppb, respectively. The lower values for fumonisin and deoxynivalenol from the Romer laboratory are difficult to explain but are most likely explained by sampling variation. Singh and Mehta (2020) reported that there is variation in reproducibility and repeatability in the analysis of mycotoxins via gas chromatography-mass spectrometry and immunosensor. However, both NDSU (original study) and Romer Labs (current study) utilized liquid chromatography-tandem mass spectrometry for which Singh et al. (2020) reported good sensitivity and confirmation of analysis. The differences between the NDSU and the Romer analyses would be due to a combination of lab-lab variation as well as the sampling variation.

**Table 2.** Mycotoxin composition of the diets^1^^,^^2^

Mycotoxin, ppb	DL	Clean corn	Corn fines	Diet 1 (0% fines)	Diet 2 (20% fines)	Diet 3 (40% fines)	Diet 4 (60% fines)	Diet 5 (60% fines + B)	Diet 6 (60% fines + BPF)
Fumonisin B_1_	100	300	11,400	600	3100	5600	8500	6600	5000
Fumonisin B_2_	100	100	3800	100	900	1700	2300	2100	1600
Fumonisin B_3_	100	<100	900	<100	200	400	600	500	400
Zearalenone	51.7	65	1626	63	281	758	774	631	708
Deoxynivalenol	100	770	2997	401	986	2328	2490	2275	2271

^1^Additional mycotoxins assayed which were below the detection level (DL) were: aflatoxin B1 (DL-1.3 ppb), aflatoxin B2 (DL-1.2 ppb), aflatoxin G1 (DL-1.1 ppb), aflatoxin G2 (DL-1.6 ppb), HT-2 toxin (DL-100 ppb), T2 toxin (DL-100 ppb), diacetoxyscirpenol (DL-100 ppb), ochratoxin A (DL-1.1 ppb), acetyldeoxynivalenol (DL-100 ppb), nivalenol (DL-100 ppb), fusarenon X (DL-100 ppb), neosolaniol (DL-100 ppb). The unit of ppb = parts per billion or µg/kg.

^2^Samples analyzed at Romer labs (Union, MO).

The mycotoxin concentration of the fines from Romer Labs (Union, MO) is listed in Table 2. The inclusion levels in the diets are greater than Paczosa et al. (2025) where no performance effects were observed at a level of 20% inclusion. Diet 4 had 60% fines, replacing all corn with corn fines. It should be noted that the DON and FUM concentrations of Diet 4 exceeded that which would result from using a feedstuff containing mycotoxin levels at the FDA guidance (fumonisins) and advisory (deoxynivalenol) levels (FDA, 2001, 2010). Diets 5 and 6 were mixed by taking a portion of Diet 4 and adding the two possible mitigants. Diets 5 and 6 had lower analyzed concentration levels of mycotoxins than Diet 4 but this is most likely due to sampling and laboratory analytical variation because Diets 5 and 6 were not made independently but were simply made by a slight dilution of the same batch of Diet 4 thereby obviating any potential for mixing-related errors. As an additional observation relative to the differences across the diets, as corn fines were added to the diet, there was in increase in the protein, Ca and P content of the diets. This difference across diets, when diets are already formulated to meet daily nutrient needs of the pigs, would only be expected to have either no effect on performance or to possibly improve performance. Therefore, any detriment in performance that may occur across Diets 1 to 4 would be a result of the mycotoxins in those diets.

### Performance

Increasing the mycotoxin-containing corn fines resulted in a linear decrease (*P* = 0.01) in ADG for Diets 1 to 4 for weeks 1–3 (Table 3) with values for Diets 1, 2, 3, and 4 of 0.44, 0.44, 0.43, and 0.39 kg/d, respectively. However, only the highest level of fines was detrimental. Weeks 4–6 also showed a tendency (*P* < 0.10) of the fines to reduce ADG resulting in a linear reduction in the cumulative performance (*P* < 0.03) for weeks 1–6 (the duration of experimental diet exposure). Throughout the duration of the experimental period where pigs were fed contaminated diets, pigs on the 0% fines diet had an ADG of 0.62 kg/day while the pigs on the 60% diet had a reduced ADG of 0.55 kg/day. This reduction in ADG resulted in a linear decrease in BW at weeks 3 and 6 due to the inclusion of fines. At the start of the experiment, the pig allotment required all diets to have similar BW (all within 0.02 kg). Between Diets 1 and 4, there was a difference in weight at week 3 of 1.18 kg and at week 6 of 2.9 kg (8.8% reduction in BW). Because the simultaneous exposure to mycotoxins can result in additive, synergistic, or antagonistic effects (Alassane-Kpembi et al., 2017), it is not possible to immediately know what percentage of the detriment resulting from the fines was caused by FUM, DON, or ZEA.

**Table 3.** Growth performance of pigs fed increasing levels of mycotoxin containing corn fines and two potential mitigants^1^^,^^2^

Diet:	1	2	3	4	5	6		*P*-values^2^		
Item	No fines	20% fines	40% fines	60% fines	60% fines + B	60% fines + BPF	SEM	Fines	Diet 4 vs. Diet 5	Diet 4 vs. Diet 6
Body weight, kg										
Week 0	6.41	6.40	6.39	6.40	6.40	6.39	0.008	0.46	0.71	0.18
Week 3	16.16^a^	15.98^a^	15.76^a^	14.98^b^	14.91^b^	15.74^a^	0.213	0.01^L^	0.82	0.02
Week 6	33.01^a^	32.67^a^	33.32^a^	30.11^b^	30.30^b^	31.90^ab^	0.739	0.03	0.86	0.11
Week 8	45.07	46.04	45.87	43.15	44.48	45.53	1.168	0.53	0.43	0.17
Average daily gain, kg										
Week 1–3	0.44^a^	0.44^a^	0.43^a^	0.39^b^	0.39^b^	0.43^a^	0.010	0.01^L^	0.83	0.02
Week 4–6	0.80^ab^	0.80^ab^	0.83^a^	0.72^b^	0.73^b^	0.77^ab^	0.028	0.09	0.84	0.26
Week 1–6	0.62^a^	0.61^a^	0.63^a^	0.55^b^	0.56^b^	0.59^ab^	0.017	0.03^L^	0.83	0.09
Week 7–8	1.00^c^	1.07^abc^	1.05^bc^	1.09^abc^	1.18^a^	1.14^ab^	0.042	0.09	0.13	0.42
Week 1–8	0.70	0.72	0.72	0.67	0.69	0.71	0.021	0.52	0.42	0.16
Average daily feed intake, kg										
Week 1–3	0.70	0.68	0.66	0.68	0.75	0.67	0.071	0.94	0.48	0.89
Week 4–6	1.36	1.33	1.40	1.63	1.54	1.57	0.104	0.27	0.55	0.70
Week 1–6	1.03	0.99	1.02	1.12	1.14	1.11	0.077	0.67	0.90	0.89
Week 7–8	1.91	2.03	2.06	2.11	2.13	2.07	0.057	0.16^L^	0.81	0.67
Week 1–8	1.22	1.19	1.24	1.31	1.35	1.32	0.067	0.51	0.62	0.88
Gain:feed ratio										
Week 1–3	0.63	0.64	0.65	0.62	0.56	0.64	0.043	0.65	0.29	0.77
Week 4–6	0.59^a^	0.60^a^	0.60^a^	0.46^b^	0.50^b^	0.49^b^	0.029	0.01^L^	0.42	0.47
Week 1–6	0.61^a^	0.62^a^	0.62^a^	0.51^b^	0.51^b^	0.54^ab^	0.029	0.03	0.86	0.48
Week 7–8	0.52	0.53	0.51	0.52	0.56	0.55	0.014	0.17	0.07	0.12
Week 1–8	0.58^abc^	0.61^a^	0.58^ab^	0.52^c^	0.53^bc^	0.54^bc^	0.020	0.04^L^	0.84	0.47

^1^Five pens of five pigs/pen per treatment mean. Week 1 to 6 pigs were fed their respective treatment diets; week 7 to 8 all pigs received a common corn-soybean meal diet.

^2^Seperation of means conducted when fines *P <* 0.10.

^a,b,c^Means within a row without a common superscript differ (*P *< 0.05).

^L^Linear effect for dietary inclusion of fines (Diets 1 to 4).

Average daily feed intake measurements were also taken and are provided in Table 3 but show an increase in feed “intake” as dietary fines increase. This increase in feed usage is not all actual feed consumption, because the pig caretakers noted feed was being wasted into the pit. Since the values for feed disappearance do not represent actual ADFI, those values also cannot be used to calculate an accurate biological G:F ratio.

Average daily gain values agree with Reddy et al. (2018) who reported a decrease in ADG when pigs were exposed to diets containing 8,000 ppb DON for 28 d. Holanda and Kim (2021) reported an 8.8% loss in BW, 8.9% loss in ADG, 10.7% loss in ADFI, and a 3.0% increase in G:F with a dietary supplementation of 1,000 ppb DON for 21 to 48 d but no effect on these growth measures with the addition of 0 to 14,000 ppb fumonisins. Concentrations of FB_1_ from 200 to 15,000 ppb for 6 wk resulted in a linear reduction in those same performance parameters (ADG, ADFI and G:F ratio) (Gbore, 2009). However, Bracarense et al. (2012) reported that diets containing 3,000 and 6,000 ppb of DON and fumonisin resulted in no adverse effects on ADG. While the reported results are extremely variable, the levels of mycotoxin-containing corn fines in the current experiment did result in a detriment in ADG which does allow the two possible mitigants to be evaluated for their ability to ameliorate the negative effects.

Measurements were taken for week 7–8 to investigate possible compensatory growth after pigs challenged with mycotoxins received a common diet. The ADG showed a tendency (*P* = 0.05) for pigs that were originally on diets with increasing fines to have an increased ADG. There was also a decreased BW at week 6 due to fines (*P* = 0.03), but at week 8 there was no BW difference. During Weeks 6–8, there was no observed feed wastage. During this period, pigs showed a linear increase in ADFI because of the increased fines in their respective diets during the period when they had contaminated diets. The pigs on the diets containing corn fines showed possible compensatory growth with no inhibition caused by the mycotoxins in the previous diet.

### Possible Mitigants

Boron, as sodium tetraborate decahydrate, was a possible mitigant added to 60% corn fines at 40 ppm (Diet 5) based upon the observations of Taranu et al. (2011) who reported that the addition of boron as calcium fructoborate ameliorated the reduction in performance caused by fusarium mycotoxins. Taranu et al. (2011), in a small study with 4 pigs per diet, fed diets contaminated with 1,790 ppb DON, 20 ppb ZEA, 0.6 ppb fumonisins, and 90 ppb T-2/H-2 toxin for 24 d and observed a 36% reduction in ADG with 84% of that reduction recovered with the organic boron product. The comparison of Diet 4 to Diet 5 in the present study indicates that the inorganic sodium tetraborate decahydrate does not alleviate the negative effects that corn fines had on BW and ADG in this experiment. The other mitigant added to create Diet 6 was 60% fines and 0.25% Biofix Plus with FUMzyme (BPF; dsm-firmenich, Plainsboro, NJ). The addition of FUMzyme resulted in a difference from Diet 4 in week 3 BW and in weeks 1–3 ADG (*P* = 0.02 and 0.02, respectively) with a tendency to be different for weeks 1–6 (*P* < 0.10). Also, it can be noted that the performance values for Diet 6 BW and ADG were not significantly different than those from Diet 1. For weeks 1–6, Diet 6 was able to recover 57% of the lost ADG caused by the addition of mycotoxin-containing corn fines. The mitigant contains FUMzyme, a fumonisin esterase that degrades fumonisins to non-toxic forms (EFSA, 2014). A study on Biofix Select Pro (without FUMzyme; DSM Nutritional Products, Parsippany, NJ) with the addition of 30,000 ppb FUM for 28 d resulted in a 78% recovery of the G:F ratio and 100% ADG recovery of the detriment caused by the FUM. A second experiment with a higher level of 60,000 ppb FUM for 14 d also found Biofix Select Pro to ameliorate the lost ADG and G:F (86% and 93%, respectively) detriment caused by FUM (Rao et al., 2020a).

To quantitively measure the effect FUMzyme has on fumonisin toxicosis, sphinganine (SA) and sphingosine (SO) biomarkers are measured. Fumonisin toxicosis results in competitive inhibition with sphinganine (SA) because of their similar structures. This disrupts the de novo synthesis of complex sphingolipid biosynthesis by competing for ceramide synthase. This disruption causes an increase in the SA:SO ratio because SA and SO are not being converted to sphingolipids by ceramide transferase. The ratio increases because SA concentration increases first and then SO usually increases once sufficient cell injury triggers membrane degradation. Depending on the duration of fumonisin toxicosis, synthesis of new sphingolipids can be blocked entirely depleting the total mass of cellular sphingolipids (Merrill et al., 2001). These effects on SA and SO metabolism being blocked result in a reliable biomarker of fumonisin toxicosis. Table 4 shows the effect on SA and SO when increasing levels of (fumonisin containing) fines were added to the diet. The overall SA values show a linear increase (*P* = 0.004) in concentration as fines increased from Diets 1 to 4. The SA concentration for Diet 1 was measured at 7,062 pmol and Diet 4 analyzed for a concentration of 151,773 pmol, the addition of FUMzyme ameliorated 93% of the increased SA caused by FUM. The SO numbers increased at a lesser rate than the SA numbers and show no significance. The high rate of increase for SA and the low rate of SO caused the SA:SO ratio to increase. This caused a linear increase (Diets 1 to 4; *P* = 0.001) in ratio associated with increased corn fines. Diet 1 analyzed for a ratio of 0.022 while Diet 4 analyzed for 0.425 and Diet 6 relieved 95% of the increased ratio. This SA: SO ratio increase was similarly seen (Rao et al., 2020b) when pigs weighing 8.9 kg were fed a dietary treatment of 7,200 ppb fumonisin for 28 d (SA:SO = 0.55); in the same experiment, as the fumonisin increased to a concentration of 35,100 ppb for 14 d, the ratio was observed to increase up to 1.58. This supports the idea that SA:SO as a biomarker is sensitive and reliable. While we previously stated that it is not possible to immediately know what percentage of the detriment, resulting from the fines, was caused by the individual mycotoxins FUM, DON, or ZEA, it can be estimated. If it is assumed that changes in the SA:SO ratio or the SA values are related to changes in growth and if it is assumed that a 95% recovery of the SA:SO biomarker values also represents a 95% recovery of the growth detriment, then the percentage of growth rate detriment in this study associated with the FUM would be about 60% with the remaining 40% loss of daily gain due to the DON and ZEA being about 40% (growth rate detriment from FUM = 0.62 to 0.55 = 0.07; growth rate recovery = 0.59 to 0.55 = 0.04; percentage recovery of ADG with the mitigant = 0.04/0.07 = 57.1%; probable loss of ADG associated with FUM = 57.1/**0**.95 = 60.2%).

**Table 4.** Sphinganine and sphingosine levels in the serum, at week 6, of pigs fed varying levels of mycotoxin containing corn fines and a potential mitigant^1^^,^^2^^,^^3^^,^^4^

		Diet						*P*-values		
		1	2	3	4	6	SEM	L	Q	4 vs. 6
Item	n	No fines	20% fines	40% fines	60% fines	60% fines + BPF				
SA, pmol	19	7,062^b^	59,204^b^	81,757^ab^	151,773^a^	15,926^b^	31,876	0.004	0.565	0.005
M	10	4,867	79,007	111,676	77,923	12,786	36,807			
F	9	9,258	39,403	51,838	225,623	19,066	52,054			
SO, pmol	19	217,185	254,498	300,960	331,312	291,516	87,330	0.259	0.945	0.702
M	10	226,956	262,300	432,818	256,481	350,804	100,840			
F	9	207,414	246,697	169,103	406,142	232,228	142,610			
SA:SO	19	0.042^d^	0.220^bc^	0.288^ab^	0.425^a^	0.061^cd^	0.067	0.001	0.524	0.001
M	10	0.022	0.272	0.269	0.299	0.044	0.078			
F	9	0.063	0.168	0.307	0.551	0.077	0.110			

^1^Serum was analyzed at week 6 for selected pigs.

^2^Means represent 4 pigs for treatment 1, 4 pigs for treatment 2, 3 pigs for treatment 3, 4 pigs for treatment 4, and 4 pigs for treatment 6. M = males, F = females.

^3^SA = sphinganine, SO = sphingosine.

^4^Statistical analysis revealed no sex or sex*treatment interaction (*P* > 0.10).

^a,b,c,d^Means within a row without a common superscript differ (*P *< 0.05).

### Clinical Chemistry

Other physiological parameters investigated during this study were serum clinical chemistry analytes (Tables 5–7). Mean sodium and chloride values were linearly decreased for Diets 1 to 4. The other measured mineral analytes (Table 5) did not show a response to fines or the addition of the two possible mitigants.

**Table 5.** Mineral analyte clinical chemistry for pigs fed varying levels of mycotoxin containing corn fines^1^^,^^2^^,^^5^

	Diet:	1	2	3	4	5	6		*P*-values	
Item	Merck Reference range	0% fines	20% fines	40% fines	60% fines	60% fines + B	60% fines + BPF	SEM^3^	Diet 4 vs. Diet 5	Diet 4 vs. Diet 6
Sodium, mmol/L	139–153	144.1	143.2	143.4	141.7	143.3	142.7	0.69^L^	0.10	0.29
Light pig		143.0	14.7	143.7	140.9	142.6	142.3	1.21	0.30	-
Median pig		144.5	143.6	143.8	140.5	143.0	143.8	1.69	0.27	-
Heavy pig		144.7	142.9	142.7	143.8	144.4	142.0	0.91	0.10	-
Potassium, mmol/L	4.4–6.5	6.31	6.30	5.87	6.01	5.95	5.93	0.19	0.82	0.74
Light pig		6.30	6.52	5.73	6.13	5.51	5.92	0.34	-	-
Median pig		6.50	6.70	6.16	5.73	5.86	6.23	0.33^L^	-	-
Heavy pig		6.18	5.81	6.08	6.17	6.37	5.64	0.34	-	-
Chloride^4^, mmol/L	97–106	103.6	102.8	102.2	101.8	102.4	102.7	0.62^L^	0.44	0.28
Light pig		103.6	101.3	100.2	100.0	102.3	102.3	1.47	-	-
Median pig		104.8	102.8	103.8	101.4	102.6	102.9	1.09^L^	-	-
Heavy pig		102.6	103.8	102.0	103.9	102.8	102.8	0.87	-	-
Calcium, mg/dL	9.3–11.5	11.66	11.58	11.55	11.43	11.34	11.38	0.13	0.53	0.79
Light pig		11.45	11.65	11.59	11.39	11.08	11.36	0.22	-	-
Median pig		11.86	11.57	11.64	11.32	11.23	11.63	0.33	-	-
Heavy pig		11.68	11.53	11.39	11.57	11.53	11.16	0.23	-	-
Phosphorus, mg/dL	5.5–9.3	10.22	10.35	10.50	10.48	10.77	11.07	0.24	0.37	0.06
Light pig		10.23	10.36	10.19	10.06	10.11	10.98	0.54	-	0.21
Median pig		10.52	10.34	10.74	10.39	11.09	11.19	0.46	-	0.13
Heavy pig		9.91	10.38	10.68	10.99	11.11	11.03	0.57	-	0.95
Magnesium, mg/dL	2.3–3.5	2.76	2.71	2.79	2.70	2.76	2.66	0.08	0.60	0.72
Light pig		2.75	3.05	2.90	2.87	2.64	2.79	0.14	-	-
Median pig		2.76	2.61	2.70	2.70	2.80	2.60	0.14	-	-
Heavy pig		2.78	2.56	2.82	2.53	2.88	2.58	0.18	-	-

^1^Each mean analyte value represents the mean of 5 replicates (3 pigs/pen) for blood collected on week 6.

^2^In each pen, the light, median, and heavy pig were selected on week 3 for blood collection.

^3^SEM represent highest value.

^4^Light, median, and heavy pig values differ (*P *< 0.05).

^5^Mean separation done if treatment *P <* 0.10.

^a,b^Means within rows and columns without a common superscript under their respective analyte differ (*P *< 0.05).

^L^Linear effect for dietary inclusion of fines (Diets 1 to 4; *P *< 0.05).

Protein and energy analytes (Table 6) were also measured to analyze a possible pattern with the inclusion of fines and two possible mitigants. Albumin was linearly decreased for Diets 1 to 4, with the median pig showing a similar response. Cholesterol decreased as the inclusion rate of fines increased (*P* = 0.02), but the pattern of the response differed among the pigs of different weight groups. The addition of BPF was significant (*P* = 0.03), recovering over 100% of the cholesterol change due to fines.

**Table 6.** Protein and energy analyte clinical chemistry for pigs fed varying levels of mycotoxin containing corn fines^1^^,^^2^^,^^5^

	Diet:	1	2	3	4	5	6		*P*-values	
Item	Merck Reference range	0% fines	20% fines	40% fines	60% fines	60% fines + B	60% fines + BPF	SEM^3^	Diet 4 vs. Diet 5	Diet 4 vs. Diet 6
Total protein, g/dL	5.8–8.3	5.45	5.36	5.42	5.26	5.26	5.25	0.08	0.99	0.90
Light pig		5.46	5.40	5.52	5.47	5.08	5.41	0.15	-	-
Median pig		5.28	5.34	5.25	5.03	5.39	5.14	0.15	-	-
Heavy pig		5.60	5.36	5.51	5.29	5.34	5.19	0.17	-	-
Albumin, g/dL	2.3–4.0	4.22	4.14	4.16	3.95	4.22	4.09	0.08^L^	0.02	0.20
Light pig		4.04	4.16	4.03	4.01	4.07	4.02	0.13	0.73	-
Median pig		4.23	4.14	4.18	3.67	4.39	4.05	0.20^L^	0.01	-
Heavy pig		4.38	4.16	4.31	4.18	4.25	4.19	0.16	0.93	-
Globulin^4^,g/dL	3.9–6.0	1.23	1.22	1.25	1.31	1.05	1.16	0.07	0.01	0.13
Light pig		1.42	1.24	1.49	1.46	1.02	1.39	0.16	0.05	-
Median pig		1.05	1.20	1.07	1.36	1.00	1.09	0.16	0.11	-
Heavy pig		1.23	1.20	1.20	1.12	1.09	1.00	0.11	0.35	-
A/G ratio^4^		3.57	3.49	3.53	3.24	4.17	3.84	0.25	0.01	0.08
Light pig		3.02	3.40	2.82	2.93	4.30	3.12	0.48	0.04	0.77
Median pig		4.08	3.57	4.08	2.98	4.24	4.16	0.66	0.15	0.12
Heavy pig		3.61	3.61	3.63	3.82	3.93	4.23	0.42	0.81	0.38
Glucose, mg/dL	66–116	112.8	115.3	118.0	113.1	111.2	114.8	2.19	0.51	0.57
Light pig		112.5	119.9	115.3	113.0	110.1	116.6	4.46	-	-
Median pig		115.8	115.1	118.9	113.4	111.8	115.8	5.45	-	-
Heavy pig		110.1	111.8	118.1	113.0	111.3	111.8	5.26	-	-
Cholesterol^6^, mg/dL	81–134	84.86	74.96	80.26	76.67	74.14	85.42	3.07	0.54	0.03
Light pig		79.58	67.10	84.10	73.10	78.30	91.50	5.55^Q^	-	0.02
Median pig		87.67	74.08	80.25	79.00	76.95	80.75	7.04	-	0.82
Heavy pig		87.33	80.58	69.15	77.92	69.75	84.00	4.63^L^	-	0.24

^1^Each mean analyte value represents the mean of 5 replicates (3 pigs/pen) for blood collected on Week 6.

^2^In each pen, the light, median, and heavy pig were selected on week 3 for blood collection.

^3^SEM represent highest value.

^4^Light, median, and heavy pig values differ (*P *< 0.05).

^5^Mean separation done if treatment *P <* 0.10.

^6^Increasing levels of fines reduced serum cholesterol concentration (*P* = 0.02).

^L,Q^Linear and quadratic effect, respectively, for dietary inclusion of fines (Diets 1 to 4; *P *< 0.05).

Blood urea nitrogen (BUN) was reduced by the inclusion of fines (*P* = 0.048; Table 7). The inclusion of BPF returned the lost BUN serum concentration from Diet 4 to above Diet 1 (15.0, 13.6, and 16.0 mg/dL for Diets 1, 4, and 6, respectively). The other liver or kidney analytes measured show no significant difference resulting from the addition of fines or the two possible mitigants.

**Table 7.** Liver and kidney analyte clinical chemistry for pigs fed varying levels of mycotoxin containing corn fines^1^^,^^2^^,^^5^

	Diet:	1	2	3	4	5	6		*P*-values	
Item	Merck Reference range	0% fines	20% fines	40% fines	60% fines	60% fines + B	60% fines + BPF	SEM^3^	Diet 4 vs. Diet 5	Diet 4 vs. Diet 6
BUN^6^, mg/dL	8.2–25	15.0	13.2	13.0	13.6	14.3	16.0	0.79	0.54	0.03
Light pig		16.5	12.8	11.6	12.6	13.3	15.0	1.37^L^	-	0.19
Median pig		15.7	12.6	13.7	13.2	15.5	15.1	1.75	-	0.33
Heavy pig		12.8^b^	14.0^ab^	14.1^ab^	15.2^ab^	15.0^ab^	17.9^b^	1.83	-	0.18
Creatinine^4^, mg/dL	0.8–2.3	1.17	1.11	1.02	1.02	1.06	1.07	0.04^L^	0.53	0.34
Light pig		1.31	1.17	1.07	1.13	1.04	1.12	0.10	-	-
Median pig		1.08	1.15	1.00	0.98	1.09	1.07	0.05^L,Q^	-	-
Heavy pig		1.13^a^	1.01^ab^	1.01^ab^	0.96^b^	1.06^ab^	1.03^ab^	0.05^L^	-	-
BUN/Creatinine ratio^4^, mg/dL		12.9	12.0	12.8	13.9	13.9	14.9	0.81	0.99	0.36
Light pig		12.8	11.1	11.4	11.9	13.1	13.4	1.71	-	-
Median pig		14.4	11.1	13.3	13.8	14.2	14.0	1.66	-	-
Heavy pig		11.5	13.6	13.9	16.0	14.9	17.3	1.59^L^	-	-
Total bilirubin, mg/dL	0–0.5	0.27	0.27	0.29	0.28	0.26	0.30	0.03	0.55	0.62
Light pig		0.23	0.32	0.38	0.29	0.24	0.27	0.05	-	-
Median pig		0.29	0.28	0.28	0.27	0.29	0.31	0.07	-	-
Heavy pig		0.28	0.23	0.25	0.29	0.25	0.33	0.03	-	-
Alkaline phosphatase, U/L	41–176	281.2	275.5	279.5	250.1	289.5	258.7	26.96	0.28	0.81
Light pig		205.5	305.6	287.0	251.0	291.7	228.7	43.68	-	-
Median pig		331.0	243.8	307.1	255.4	252.8	272.4	73.13	-	-
Heavy pig		307.0	300.5	252.6	243.8	302.8	274.9	45.78	-	-
SGOT/AST, U/L	15–55	25.3^b^	31.5^ab^	23.7^b^	37.5^a^	31.4^ab^	30.6^ab^	4.70	0.34	0.26
Light pig		22.3^c^	55.2^a^	29.0^bc^	51.8^ab^	28.8^bc^	24.8^c^	8.49	-	-
Median pig		29.0	23.5	21.2	30.4	30.9	29.5	6.05	-	-
Heavy pig		24.7	23.9	26.7	30.3	31.4	37.5	7.50	-	-

^1^Each mean analyte value represents the mean of 5 replicates (3 pigs/pen) for blood collected on week 6.

^2^In each pen, the light, median, and heavy pig were selected on week 3 for blood collection.

^3^SEM represent highest value.

^4^Light, median, and heavy pig values differ (*P *< 0.05).

^5^Mean separation done if treatment *P <* 0.10.

^6^Increasing levels of fines reduced serum blood urea nitrogen concentration (*P* = 0.048).

^a,b,c^Means with different superscripts within rows under their respective analyte differ (*P *< 0.05) if.

^L,Q^Linear and quadratic effect, respectively, for dietary inclusion of fines (Diets 1 to 4; *P *< 0.05) if treatment *P <* 0.10.

Compared to Paczosa et al. (2025), where the maximum addition of 20% mycotoxin-containing corn fines to the diet significantly decreased serum sodium and cholesterol levels and significantly increased serum glucose and magnesium levels, only cholesterol responded similarly due to the addition of fines. This indicates that measuring these specific serum analytes, except cholesterol, may not be a consistent biomarker of these particular mycotoxins in the diet. Yet, while the increased concentration of cholesterol observed herein is consistent with that observed by Paczosa et al. (2025), which was conducted in the same facilities with similar genetics and the same batch of fines, it does not agree with the information available in the literature. The literature states that liver damage caused by mycotoxins will reduce blood serum cholesterol (Holanda and Kim, 2021). Additionally, it should be noted that even in the few instances where statistically significant differences were observed in the analytes provided in Tables 5–7, when the values are compared to the Merck reference ranges, the changes observed are small and usually within the reference range. Because this current study and Paczosa et al. (2025) did not show a significant effect of the mycotoxins from the fines on the liver analytes, it is possible that the level of mycotoxins obtained from the fines was not high enough or the period of feeding long enough to induce changes in the analytes.

## CONCLUSION

In conclusion, as mycotoxins increased, pigs exhibited adverse effects in ADG and BW, but the additive Biofix Plus with FUMzyme ameliorated a portion of these effects. Further, the improved performance from weeks 7–8 for all pigs on all diets that had fines does show the importance of feeding clean corn to optimize ADG, ADFI, and gain/feed ratio. To measure possible fumonisin toxicosis, this experiment supports the SA: SO ratio as an accurate biomarker. Lastly, more work needs to be done to investigate further the interactions of these particular mycotoxins and how the serum clinical chemistry will respond.

## References

[CIT0001] Ag Guide. 2020. Guide for the care and use of agricultural animals in research and teaching. 4th ed. Champaign (IL): Federation of Animal Science Societies.

[CIT0002] Alassane-Kpembi, I., G. Schatzmayr, I. Taranu, D. Marin, O. Puel, and I. P. Oswald. 2017. Mycotoxins co-contamination: Methodological aspects and biological relevance of combined toxicity studies. Crit. Rev. Food Sci. Nutr. 57:3489–3507. doi: https://doi.org/10.1080/10408398.2016.1140632 26918653

[CIT0003] AOAC Int. 2006. Official methods of analysis of AOAC Int. 18th ed. Rev. 1. Gaithersburg (MD): AOAC Int.

[CIT0004] Bracarense, A. -P. F. L., J. Lucioli, B. Grenier, G. Drociunas Pacheco, W. -D. Moll, G. Schatzmayr, and I. P. Oswald. 2012. Chronic ingestion of deoxynivalenol and fumonisin, alone or in interaction, induces morphological and immunological changes in the intestine of piglets. Brit. J. Nutr. 107:1776–1786. doi: https://doi.org/10.1017/S0007114511004946 21936967

[CIT0005] Cargill. 2022. Cargill World Mycotoxin Report. [accessed August 4, 2023]. https://www.cargill.com/feedingintelligence/2022-cargill-world-mycotoxin-report

[CIT0006] dsm. 2024. Mycotoxin survey in North America: June 2024 Update. [accessed November 12, 2024]. 35 - Mycotoxin Survey Monthly Update: June 2024

[CIT0007] EFSA. 2014. Scientific opinion on the safety and efficacy of fumonisin esterase (FUMzyme®) as a technological feed additive for pigs. EFSA J. 12:3667. doi: https://doi.org/10.2903/j.efsa.2014.3667 PMC13159837 42125390

[CIT0009] FDA. 2001. Guidance for industry: fumonisin levels in human foods and animal feeds. [accessed February 5, 2025]. https://www.fda.gov/regulatory-information/search-fda-guidance-documents/guidance-industry-fumonisin-levels-human-foods-and-animal-feeds.

[CIT0008] FDA. 2010. Guidance for industry and FDA: advisory levels for deoxynivalenol (DON) in finished wheat products for human consumption and grains and grain by-products used for animal feed. [accessed February 5, 2025]. https://www.fda.gov/regulatory-information/search-fda-guidance-documents/guidance-industry-and-fda-advisory-levels-deoxynivalenol-don-finished-wheat-products-human.

[CIT0010] Frobose, H. L., E. D. Fruge, M. D. Tokach, E. L. Hansen, J. M. DeRouchey, S. S. Dritz, R. D. Goodband, and J. L. Nelssen. 2015. The effects of deoxynivalenol-contaminated corn dried distillers grains with solubles in nursery pig diets and potential for mitigation by commercially available feed additives. J. Anim. Sci. 93:1074–1088. doi: https://doi.org/10.2527/jas.2013-6883 26020884

[CIT0011] Gbore, F. A. 2009. Growth performance and puberty attainment in growing pigs fed dietary fumonisin B_1_. J. Anim. Physiol. Anim. Nutr. (Berl) 93:761–767. doi: https://doi.org/10.1111/j.1439-0396.2008.00866.x 19175462

[CIT0012] Holanda, D. M., and S. W. Kim. 2021. Mycotoxin occurrence, toxicity, and detoxifying agents in pig production with an emphasis on deoxynivalenol. Toxins (Basel) 13:171. doi: https://doi.org/10.3390/toxins13020171 33672250 PMC7927007

[CIT0026] Hsiao, S. H., M. E. Tumbleson, P. D. Constable, and W. M. Haschek. 2007. Use of formalin-fixed tissues to determine fumonisin B1-induced sphingolipid alterations in swine. J Vet Diagn Invest. 19:425–430. doi: https://doi.org/10.1177/104063870701900417 17609357

[CIT0013] Kim, B. G., and M. D. Lindemann. 2007. A spreadsheet method for experimental animal allotment. J. Anim. Sci. 85:112. [accessed October 25, 2022]. https://afs.ca.uky.edu/swine/computer-programs/experimental-animal-allotment-program

[CIT0014] Lindemann, M. D., D. J. Blodgett, E. T. Kornegay, and G. G. Schurig. 1993. Potential ameliorators of aflatoxicosis in weanling/growing swine. J. Anim. Sci. 71:171–178. doi: https://doi.org/10.2527/1993.711171x 8384193

[CIT0015] Merrill, A. H., Jr., M. C. Sullards, E. Wang, K. A. Voss, and R. T. Riley. 2001. Sphingolipid metabolism: roles in signal transduction and disruption by fumonisins. Environ. Health Perspect. 109:283–289. doi: https://doi.org/10.1289/ehp.01109s2283 PMC1240677 11359697

[CIT0025] National Research Council (NRC). 2012. Nutrient requirements of swine. 11th Revised ed. Washington (DC): The National Academies Press.

[CIT0016] Paczosa, D. B., T. B. Chevalier, S. A. Adedokun, L. Zheng, and M. D. Lindemann. 2025. Effects of feeding varying levels of mycotoxin-containing corn fines on diet choice and growth performance of nursery pigs. Transl. Anim. Sci. doi:10.1093/tas/txaf015 PMC11879404 40041717

[CIT0017] Rao, Z. X., M. D. Tokach, S. S. Dritz, J. C. Woodworth, J. M. DeRouchey, R. D. Goodband, and H. I. Calderon. 2020a. Efficacy of commercial products on nursery pig growth performance fed diets with fumonisin contaminated corn. Transl. Anim. Sci. 4:txaa217. doi: https://doi.org/10.1093/tas/txaa217 33409469 PMC7771004

[CIT0018] Rao, Z. X., M. D. Tokach, J. C. Woodworth, J. M. DeRouchey, R. D. Goodband, H. I. Calderón, and S. S. Dritz. 2020b. Effects of fumonisin-contaminated corn on growth performance of 9 to 28 kg nursery pigs. Toxins (Basel) 12:604. doi: https://doi.org/10.3390/toxins12090604 32961935 PMC7551907

[CIT0019] Reddy, K. E., J. Song, H. -J. Lee, M. Kim, D. -W. Kim, H. J. Jung, B. Kim, Y. Lee, D. Yu, D. -W. Kim, et al 2018. Effects of high levels of deoxynivalenol and zearalenone on growth performance, and hematological and immunological parameters in pigs. Toxins 10:114. doi: https://doi.org/10.3390/toxins10030114 29518941 PMC5869402

[CIT0020] Schell, T. C., M. D. Lindemann, E. T. Kornegay, and D. J. Blodgett. 1993a. Effects of feeding aflatoxin-contaminated diets with and without clay on performance, liver function, and mineral metabolism to weanling and growing pigs. J. Anim. Sci. 71:1209–1218. doi: https://doi.org/10.2527/1993.7151209x 8099347

[CIT0021] Schell, T. C., M. D. Lindemann, E. T. Kornegay, D. J. Blodgett, and J. A. Doerr. 1993b. Effectiveness of different types of clay for reducing the detrimental effects of aflatoxin-contaminated diets on performance and serum profiles of weanling pigs. J. Anim. Sci. 71:1226–1231. doi: https://doi.org/10.2527/1993.7151226x 8099348

[CIT0022] Singh, J., and A. Mehta. 2020. Rapid and sensitive detection of mycotoxins by advanced and emerging analytical methods: a review. Food Sci. Nutr. 8:2183–2204. doi: https://doi.org/10.1002/fsn3.1474 32405376 PMC7215233

[CIT0023] Taranu, I., D. E. Marin, G. Manda, M. Motiu, I. Neagoe, C. Tabuc, M. Stancu, and M. Olteanu. 2011. Assessment of the potential of a boron–fructose additive in counteracting the toxic effect of Fusarium mycotoxins. Br. J. Nutr. 106:398–407. doi: https://doi.org/10.1017/s0007114511000341 21396141

[CIT0024] Weaver, A. C., D. M. Weaver, N. Adams, and A. Yiannikouris. 2021. Co-Occurrence of 35 mycotoxins: a seven-year survey of corn grain and corn silage in the united states. Toxins (Basel) 13:516. doi: https://doi.org/10.3390/toxins13080516 34437387 PMC8402310

